# Invasive Malaria Vector *Anopheles stephensi* Mosquitoes in Sudan, 2016–2018

**DOI:** 10.3201/eid2711.210040

**Published:** 2021-11

**Authors:** Ayman Ahmed, Patricia Pignatelli, Arwa Elaagip, Muzamil M. Abdel Hamid, Omnia Fateh Alrahman, David Weetman

**Affiliations:** Liverpool School of Tropical Medicine, Liverpool, UK (A. Ahmed, P. Pignatelli, D. Weetman);; University of Khartoum, Khartoum, Sudan (A. Ahmed, A. Elaagip, M.M. Abdel Hamid, O. Fateh Alrahman)

## Abstract

*Anopheles stephensi* mosquitoes are urban malaria vectors in Asia that have recently invaded the Horn of Africa. We detected emergence of *An. stephensi* mosquitoes in 2 noncontiguous states of eastern Sudan. Results of mitochondrial DNA sequencing suggest the possibility of distinct invasions, potentially from a neighboring country.

*Anopheles stephensi* mosquitoes are efficient vectors of *Plasmodium vivax* and *P. falciparum*. Their native range centers on the Indian subcontinent, from which they are increasingly expanding their geographic distribution (*1*). Recent establishment in Ethiopia (*2*) and Djibouti (*3*) is especially worrying. We document the emergence of *An. stephensi* mosquitoes in Sudan.

Among study sites in a study originally investigating insecticide resistance in the dominant malaria vector in Sudan, *Anopheles arabiensis* mosquitoes, we selected 12 sites in the eastern half of the country to represent the different ecologic zones (Appendix Figure 1). We collected *Anopheles* spp. larvae from all sites in 2016 and from most again in late 2017 or early 2018 (Appendix Table 1). We reared the larvae to adults, checked them morphologically, and initially identified the species as *An. gambiae* s.l. We extracted DNA from a subset for molecular identification of the species by PCR (*3*). Of these, 149 DNA samples failed to amplify when we used the standard protocol for identification of the *An. gambiae* complex, and we investigated them further. We performed mitochondrial cytochrome oxidase 1 amplification and sequencing by using the universal primers C1-J-2183 and TL2-N-3014 on the first batch (*4*); to provide conformity with other studies in East Africa, we used Folmer primers LCO1490 and HCO2198 on a second batch (*4*). To confirm species identity, we performed BLAST (https://blast.ncbi.nlm.nih.gov/Blast.cgi) searches. We supplemented sequences generated for the mosquitoes from Sudan by using the Folmer primers with sequences from other studies downloaded from GenBank, assembled them by using Clustal within MEGAX (*5*), and displayed the results as a maximum-likelihood tree with 1,000 bootstraps.

Sequence analysis demonstrated that many of the samples failing diagnostic PCR were not *An. gambiae* s.l. mosquitoes; most samples identified by BLAST were *An. stephensi* mosquitoes (Appendix Table 1). The relative frequencies of *An. stephensi* mosquito detection were similarly high (>40%) among those from the 2 Red Sea state sites, Port Sudan and Tokar, but seem much lower elsewhere; only 1 individual *An. stephensi* mosquito was detected at each site in Gedaref state, and none were detected at the other study sites (Appendix Table 1, Figure 2). Although sample site identifications were clear, year identification labels were unfortunately not preserved during sample shipment, and from these samples we cannot determine when *An. stephensi* mosquitoes were collected. However, sequencing of pools of additional samples from Tokar and Port Sudan from each collection year, which were preserved primarily for RNA analysis, confirmed their presence in both years.

Phylogenetic analysis of the sequences identified 3 haplotypes, which we named Sudan H1–3 (Figure). The most common Sudan haplotype was H3 (86%), detected at both of the Red Sea state sites (Tokar and Port Sudan); the relatively similar haplotype H2 was detected at lower frequency at Port Sudan only (Appendix Figure 1). H2 and H3 cluster with those collected in Ethiopia in 2016 (*2*) and with 2 of those collected in 2019, for which we retained the authors’ haplotype notation (*6*). H1 was detected only in Abu Alnaja (Gedaref state) and clustered within a larger clade encompassing a broad range of locations, including a haplotype detected in both Djibouti and Ethiopia (Appendix Figure 1). The sample of *An. stephensi* mosquitoes from Daim Bakur failed to amplify after use of the Folmer primers and thus was not included in the tree. The initial origins of *An. stephensi* mosquitoes are currently difficult to ascertain because of a lack of geographic resolution in the phylogeny; the exception is the highly differentiated clade of samples from Saudi Arabia and Iran. However, the haplotypes detected in Sudan are similar to 3 of the 4 detected in Ethiopia and are thus potentially consistent with spread from Ethiopia or elsewhere in the Horn of Africa. The presence of haplotypes from different states in 2 distinct clades may also indicate separate introduction sources, although wider sampling is required for confirmation.

**Figure:**
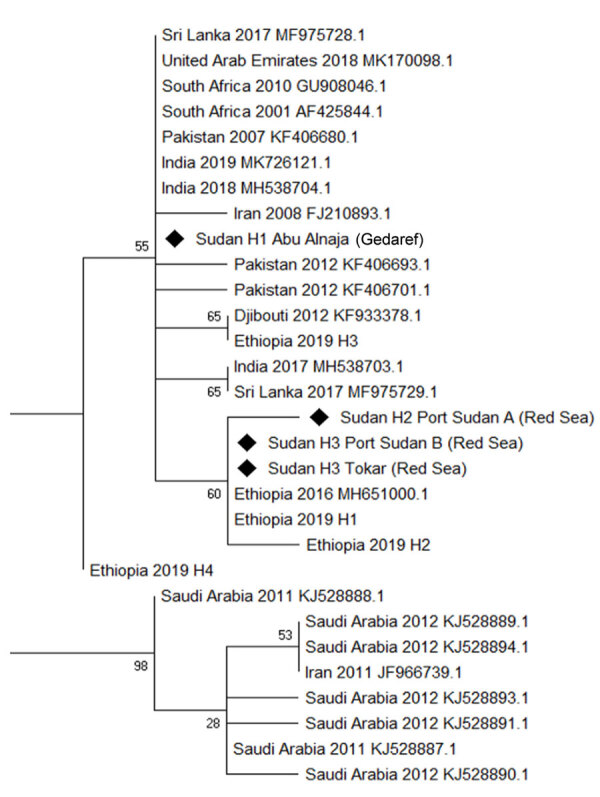
Phylogenetic analysis of *Anopheles stephensi* mosquitoes collected in Sudan, 2016–2018, and reference sequences. Maximum-likelihood tree was constructed by using mitochondrial cytochrome oxidase 1 sequences from Sudan (diamonds) and other countries from which data are available. GenBank accession numbers are provided for reference sequences.

The emergence of *An. stephensi* mosquitoes in Sudan poses substantial concern for malaria control and elimination and potentially stark predictions for urban malaria in Africa if this species should spread farther (*7*). In Sudan and throughout much of Africa, local surveillance systems, as well as knowledge and expertise, focus mainly on the mosquito vector members of the predominant *An. gambiae* complex and *An. funestus* group (*8*). Although the actual epidemiologic effects of *An. stephensi* mosquito emergence is not known, temporal coincidence of their establishment and rising malaria rates in Djibouti suggest a substantial threat (*9*). Although little is known about *An. stephensi* mosquitoes in Sudan, we identified productive breeding sites in septic tanks, manholes, and the water-storage containers used for construction purposes in cities (Appendix Figure 3); these findings correspond with reports from Ethiopia (*6*). Information about the wider and local distribution of *An. stephensi* mosquitoes in Sudan, coupled with bionomic studies, can be used to guide rational control strategies.

Appendix Supplemental methods and results for study of invasive malaria vector *Anopheles stephensi* mosquitoes in Sudan, 2016–2018.
