# Supplementary material for: Invasive Malaria Vector Anopheles stephensi Mosquitoes in Sudan, 2016–2018
Source: Emerg Infect Dis. 2021 Nov;27(11):2952–4. doi: 10.3201/eid2711.210040 (PMC8544976; doi:10.3201/eid2711.210040)
Supplement: Appendix — Supplemental methods and results for study of invasive malaria vector Anopheles stephensi mosquitoes in Sudan, 2016–2018. [file 21-0040-Techapp-s1.pdf]

Article DOI: https://doi.org/10.3201/eid2711.210400

Invasive Malaria Vector *Anopheles stephensi* Mosquitoes in Sudan, 2016–2018

Appendix

Appendix Table 1. Collection information and species proportions detected using a combination of standard PCR diagnostics and mitochondrial DNA sequencing Estimated percentages of *Anopheles stephensi* (with associated binomial confidence intervals) are calculated from positive identifications from sequence data divided by the total number of DNA samples (which were initially run in PCRs)

Location	State	Collected	Total DNA samples	PCR: positive as *An. arabiensis*	PCR: not *An. gambiae s.l.*	Total sequenced	*An. gambiae s.l.*	*An. rufipes*	*An. stephensi*	No result	Estimated % *An. stephensi*	95% Lower	95% Upper
Abu Alnaja	Gedaref	2016, 2017	25	20	5	5	2	0	1	2	4.0%	0.1%	20.4%
Daim Bakur	Gedaref	2016, 2017	25	12	13	13	4	0	1	8	4.0%	0.1%	20.4%
Sennar	Sennar	2016, 2017	25	17	8	8	2	6	0	0	0%	0%	13.7%
Um elkhair	Sennar	2016, 2017	25	25	0	0	0	0	0	0	0%	0%	13.7%
Ziedab	River Nile	2018	25	17	8	8	5	0	0	3	0%	0%	13.7%
Makarab	River Nile	2016, 2017	25	25	0	0	0	0	0	0	0%	0%	13.7%
Port Sudan	Red Sea	2016, 2018	94	41	53	53	0	0	41	12	43.6%	33.4%	54.2%
Tokar	Red Sea	2016, 2018	127	23	104	104	43	0	52	9	40.9%	32.3%	50.0%
Keryab	Khartoum	2016, 2018	25	25	0	0	0	0	0	0	0%	0%	13.7%
Trais	Khartoum	2018	25	25	0	0	0	0	0	0	0%	0%	13.7%
Keriaba	Al Gezira	2016, 2017	25	25	0	0	0	0	0	0	0%	0%	13.7%
Hajabdall	Al Gezira	2016, 2017	25	25	0	0	0	0	0	0	0%	0%	13.7%

Appendix Table 2. Present frequencies of the detected haplotype of *An. stephensi* per the study sites

Haplotype	Abu Alnaja	Port Sudan	Tokar	N (%)	GenBank accession no.
H1	1	0	0	1 (4.5%)	MW197099
H2	0	2	0	2 (9.1%)	MW197100
H3	0	12	7	19 (86.4%)	MW197101
Total	1	14	7	22 (100%)	-

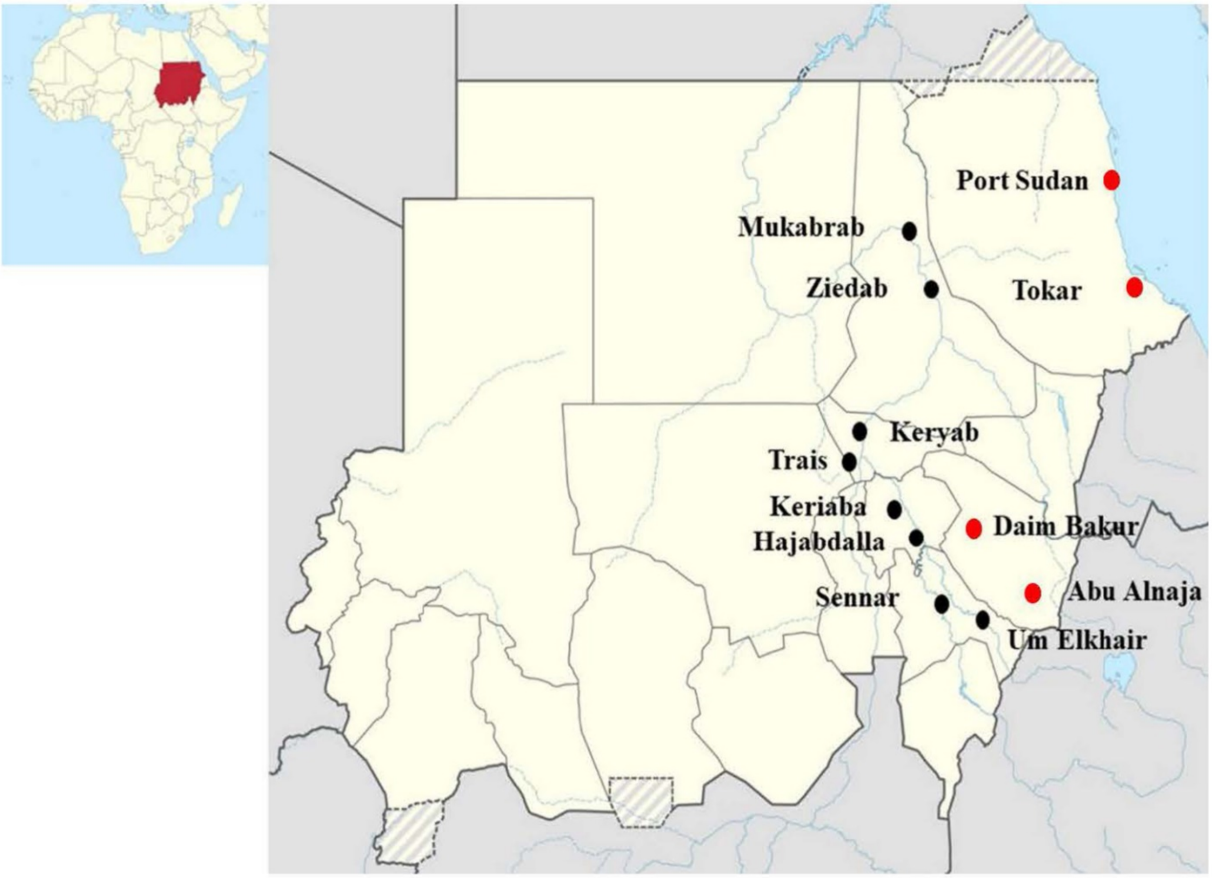

Appendix Figure 1. Location of sampling locations. Red indicates sites of *Anopheles stephensi* mosquito collection.

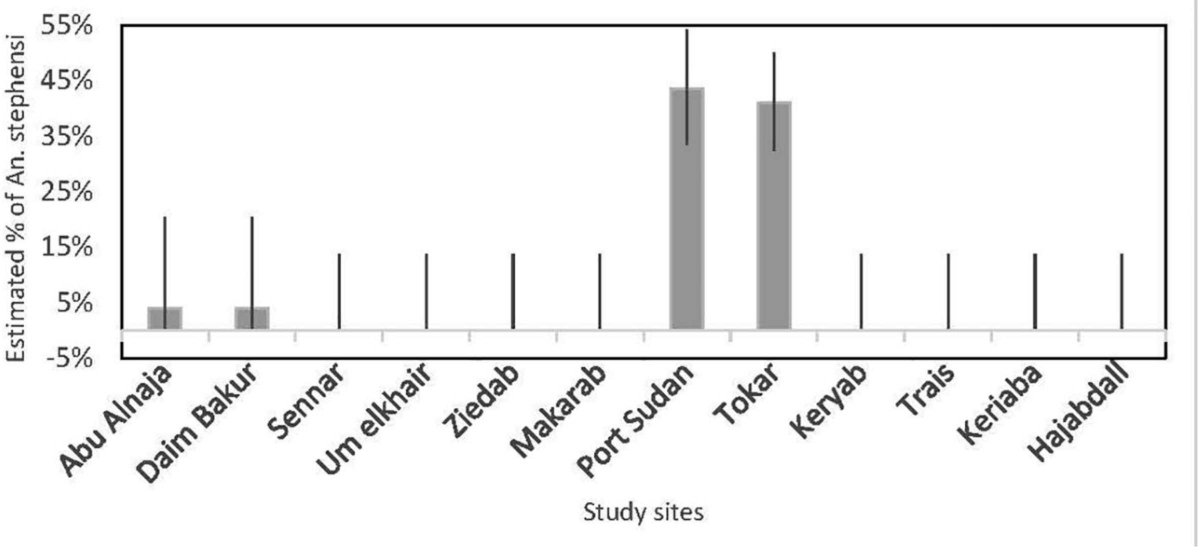

Appendix Figure 2. Percentage of *Anopheles stephensi* mosquitoes from the total collected mosquito samples per each study site.

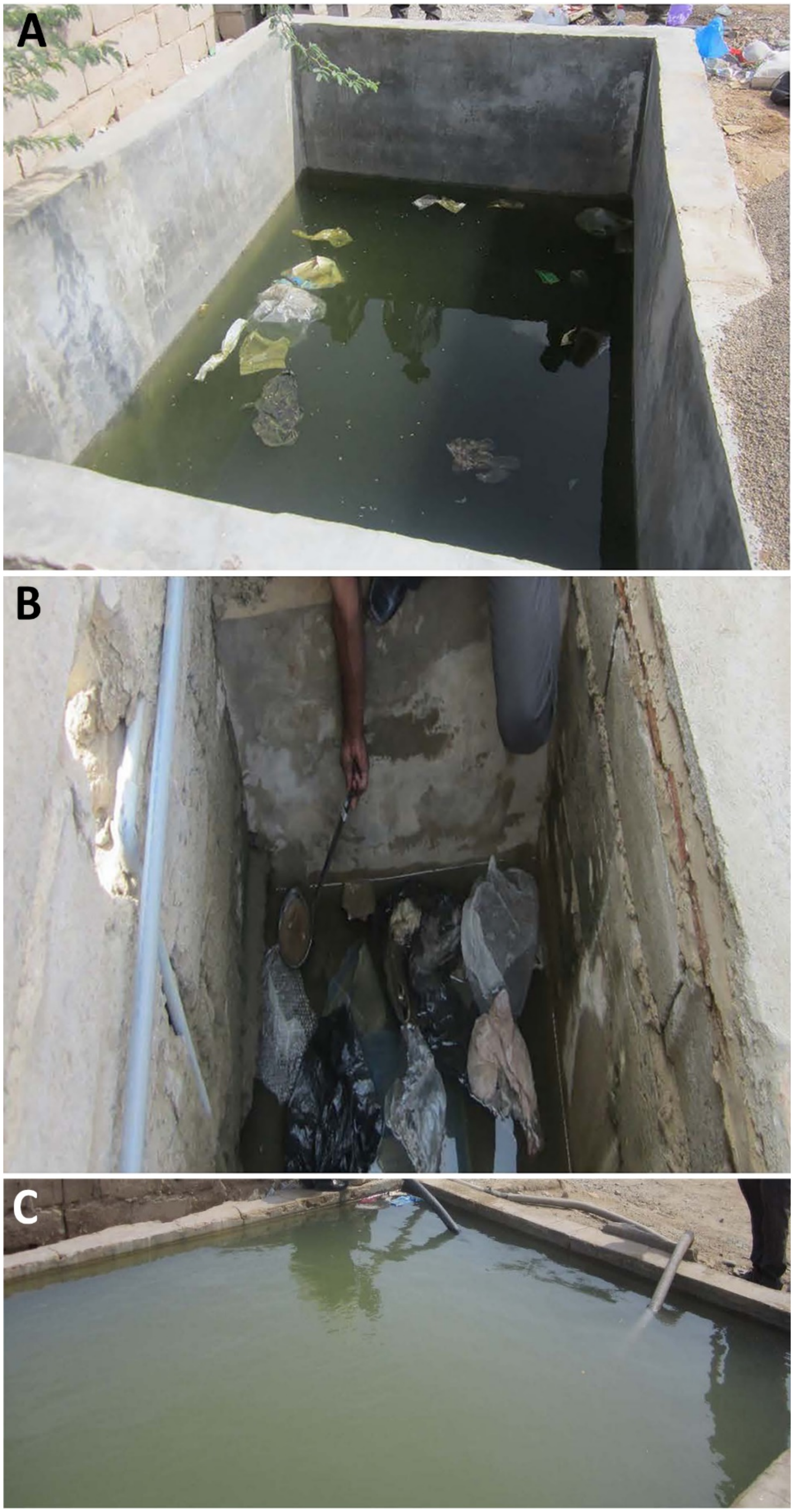

Appendix Figure 3. Types of breeding sites from which larvae of *Anopheles stephensi* mosquitoes were collected. A) Septic tank; B) manhole; C) a concrete tank for water storage used during construction.
